# Patient-Derived Design Principles for Technology-Enabled Healing at Home Following Hospital Discharge: Mixed Methods Study

**DOI:** 10.2196/72913

**Published:** 2025-08-20

**Authors:** Lindsey M Philpot, Abhinav Singla, Sagar B Dugani, Rachel E Canning, Christina M Smith, Meredith A DeZutter, Priya Ramar, Jennifer M P Hovell, Jon O Ebbert

**Affiliations:** 1 Department of Medicine, Mayo Clinic, 200 First Street SW, Rochester, MN, 55905, United States, 1 5075381882

**Keywords:** hospital discharge, technology, user-centered design, patient transitions, digital health, remote patient monitoring, ecological momentary assessments, accelerometers

## Abstract

**Background:**

As more patients transition from hospital to home for postacute care, a growing interest exists in leveraging technology to support recovery, yet a limited understanding exists on how to design these tools to align with patient and caregiver needs and preferences.

**Objective:**

This study aimed to explore the perceptions, attitudes, and beliefs of recently discharged patients to develop user-centered design principles for digital tools that support safe and effective transitions from hospital to home.

**Methods:**

A vignette-based, mixed methods survey was conducted, grounded in the technology acceptance model, to explore patient perceptions of digital tools supporting postdischarge care. A random sample of 1000 recently discharged adult patients received a survey featuring validated vignettes and technology acceptance model–informed questions, with both quantitative and qualitative items. Open-ended responses were analyzed using grounded theory to derive design principles that inform the development and implementation of patient-centered digital health tools. Quantitative items were descriptive and are summarized as counts (n) and frequencies (%).

**Results:**

Of the 967 eligible patients contacted, 116 completed the survey (response rate of 2%), with respondents having a median age of 71 (IQR 61-78) years, high rates of chronic illness, and access to smartphones (98/116, 84.5%) and in-home internet (111/116, 95.7%). Qualitative analysis revealed 6 key themes—connection to care, technical ease of use, solution usability, human connection, cost, and privacy—informing 3 patient-centered design principles focused on user experience, affordability, and transparent communication to guide future technology-supported hospital discharge interventions. Respondents reported the following factors as highly important: reassurance that a care team member would reach out if something seemed wrong (107/116, 92.2%), responsiveness to patient needs (95/116, 81.9%), ability to see their data (95/116, 81.9%), and out-of-pocket cost (94/116, 81.0%). Less important factors included duration of device use (22/116, 19.0%) and battery life (21/116, 18.0%).

**Conclusions:**

Grounded in patient perspectives, this study identified the 3 core design principles of user experience and accessibility, cost and privacy, and communication and transparency that should guide the development and implementation of digital tools to support safe, effective, and human-centered transitions from hospital to home.

## Introduction

The quality of postacute care impacts both short- and long-term outcomes for patients discharging from the hospital [1,2]. In the United States, 2 in 5 hospital discharges of Medicare patients result in admission to a postacute care facility [1], despite evidence that most patients and caregivers have a strong preference for home-based postacute care [3]. In addition to patient preference for healing at home, limitations in hospital bed availability, restricted access to postacute care facilities, the high cost of care within these settings, and endorsement for in-home programs such as Medicare’s Acute Hospital at Home have shifted rehabilitation and recovery to patient homes [4,5].

Although most patients feel relief returning home after hospital discharge, this relief is often accompanied by anxiety and confusion about managing recovery [6]. Telephone-based postdischarge outreach by primary care is recognized as a best practice for safe transitions of care and is supported by Medicare’s transitional care management services, but can be limited due to the operational burden associated with nursing time and lack of longitudinal contact through the recovery period [7]. Technology can help facilitate patient transitions from hospital care to home by addressing challenges experienced postdischarge, including continuity of care, patient adherence to therapy plans, patient and caregiver engagement, and timely escalation of needs to prevent adverse events or complications. Automated communications via standardized text messaging (eg, ecological momentary assessments) are the most widely reported upon technology, which can be used to schedule follow-up appointments, encourage treatment adherence, and monitor symptoms [7-9]. In addition to SMS-based text messaging, other active technology support for patient transitions from hospital settings to home includes remote monitoring of conditions such as acute kidney injury [10] and heart failure [11]. In these models, patients transfer daily vitals and patient-reported outcomes back to care teams, while interactive care plans guide them through discrete tasks to help stabilize a new or evolving medical condition [12,13]. Passive technology-based support, which is enabled without direct input from the patient or caregiver, is also emerging as an option to support patients transitioning from hospital care to home, but is less studied. Accelerometers and GPSs are common elements found in modern cellular phones and smartwatches and hold the potential to detect patient movement patterns, assessing for changes in physical activity levels, gait, or falls as proxies for recovery [14-16]. These passive modes of technology support are nascent and have only been studied in specific populations [14,16]. A gap exists in the scientific literature about how to design technologies to optimize patient and caregiver acceptability [9]. This will be increasingly important as intelligent monitoring systems based on Internet of Things technologies are being developed and proposed as possible health care solutions [17,18].

Hybrid models of technological connection between patients and care team members continue to evolve and be shared in the academic literature, while additional insights into implementation and scaling of successful interventions, and opportunities to address digital disparities experienced by patients, continue to emerge [7,19]. User-centered design (UCD) approaches can optimize the human experience with technologies, facilitating increased acceptance and uptake of new tools and thereby supporting implementation and scaling of successful interventions [20]. UCD focuses on a deep understanding of the needs, preferences, and barriers experienced by end users of design tools, services, and interventions addressing the needs of people [21]. Although major funding organizations and patient advocacy groups are encouraging participatory design for digital health interventions, the principles and approaches leveraging UCD relating to technology support for transitions from hospital care to home are uncommonly described in the academic literature. Design thinking can be deployed to create design principles in the build of new tools and services grounded in a patient-centered format. These design principles are critical constructs to inform product design and build, as well as implementation strategies to support adoption and sustainability.

The objective of our study was to elicit the perceptions, attitudes, and beliefs of patients recently discharged from the hospital to create design principles to enable the design and implementation of digital tools. We present our methodology, results of our vignette-based qualitative inquiry and survey-based quantitative findings, and propose a set of design principles to be used in technology design and clinical implementation.

## Methods

### Study Design, Setting, and Population

**Figure 1.**
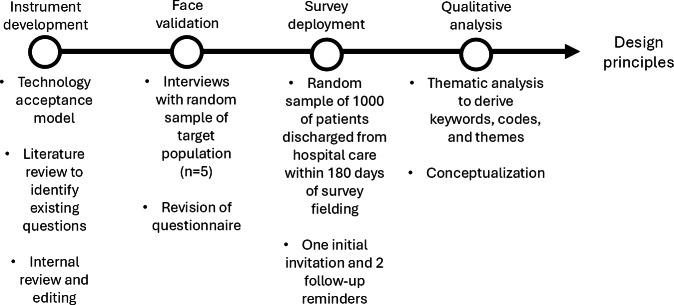
Overall approach used to delineate design principles for technology supporting patient transition from hospital to home.

This study was a vignette-based, mixed methods survey design grounded in the technology acceptance model (TAM) to investigate patient-perceived barriers and facilitators to the use of digital tools to support patient connection back to care teams following hospital discharge. The TAM is a widely used theoretical framework for understanding when, how, and why individuals use technology [22]. Incorporating TAM constructs into the development of questionnaires ensures that all aspects known to influence technology use and acceptance are assessed within the survey instrument [22]. Vignette-based studies within the qualitative research landscape allow for an in-depth understanding of patient attitudes on potential changes to their health care environment and are commonly used in the human-centered design process to facilitate the development of design principles [23]. An overview of the approach is displayed in Figure 1. A Checklist for Reporting Results of Internet E-Surveys (CHERRIES) form is provided as Multimedia Appendix 3. 

Mayo Clinic is a multispecialty medical center with campuses in Rochester, Minnesota; Jacksonville, Florida; and Phoenix, Arizona, and a health care system spanning 44 communities in southern Minnesota, western Wisconsin, and northern Iowa. The Rochester, Minnesota, campus has 2 hospitals with over 2000 beds. The target population was 1000 unique patients discharged from either of the 2 campuses in Rochester, Minnesota. To ensure the relevancy and plausibility of our findings, we focused on patients discharged within 180 days before survey distribution. A simple random sample was performed among patients aged 18 years or younger at the time of discharge, residing within the hospital’s catchment area, who had agreed to participate in research per state statute, were discharged to home, and were not deceased according to our medical records. The target sample size was 1000 patients to gain responses from at least 200 individuals, based on the 20% response rate typically observed in this population. The survey was tested, deployed, and managed by the Mayo Clinic Survey Research Center, as described in the later section “Instrument fielding.”

### Instrument Development and Testing

Instrument development involved a multistep process: development of vignettes, face validation, and survey development. Vignettes focused on 2 technologies under consideration for use by hospital practice: watch-based accelerometers intended to help hospital teams understand patient movement as a proxy for recovery, and text message–based ecological momentary assessments to check in on patients using short sets of questions. Initial vignettes were drafted by the principal investigator (LMP) in collaboration with the Mayo Clinic Center for Digital Health, responsible for the implementation of digital tools for patients as part of routine clinical care. The first review was performed by members of the research team (PR and REC) and internal medicine physicians specializing in hospital care (SBD and AS) and primary care (JOE).

Once initial vignettes were completed, they entered a phase of face validation, whereby 5 individuals fitting the criteria for our target population were interviewed one-on-one with a research team member (CMS) trained in qualitative research methods. A semistructured interview format was used, whereby each participant was provided an overview of the intent of the study and provided a printed copy of each draft vignette, as well as had the vignette read aloud by the interviewer (CMS). Each participant was asked about the clarity of the vignette through a semistructured interview. Participants were offered the option to be interviewed remotely (telephone or video) or in person, and all interviews were recorded for analysis purposes. Based on face validation, the technology vignettes were revised before launch. The interview guide is provided as Multimedia Appendix 1.

The complete survey instrument included the revised technology vignettes along with 2 open-ended questions: “What initial impressions do you have when thinking about (using an accelerometer/receiving ecologic momentary assessment) after a hospital stay?” and “What questions come to mind that you would want answered before agreeing to (wear an accelerometer/receive ecologic momentary assessment) after a hospital stay?” Following each vignette, respondents were asked to evaluate the level of importance of literature-derived or hypothesized design principles related to the use of each technology on a 5-point Likert scale. Questions grounded in the TAM that have been previously used and tested by the study team [24,25] were used to assess general use of technology such as the ability to access the internet at home or other locations, access to devices (eg, personal computers or laptops, and smartphones), and patient interest in and ability to use technology. Patient demographic factors, medical comorbidities, and patient-reported quality of life using the EuroQol 5-Dimension Scale (EQ-5D) [26] were also assessed. The survey instrument is provided as Multimedia Appendix 2.

### Instrument Fielding

The finalized instrument was built by the Mayo Clinic Survey Research Center staff within Qualtrics survey software (Qualtrics LLC) for electronic deployment, sent to patient-provided email addresses. Pilot testing of the finalized instrument led the study team to anticipate that respondents would need 20‐25 minutes to complete the survey. Based on the inclusion criteria, 1000 patients were sampled to complete the online survey on Thursday, October 31, 2024. In total, 33 invitation emails bounced upon delivery. A total of 2 reminders were sent to nonresponders on November 3 and November 8, 2024, and the survey was closed on Wednesday, November 14, 2024, with 116 total responses.

### Thematic Review and Derivation of Design Principles

Thematic review of participant-provided responses was completed using grounded theory. Open coding was performed to identify initial themes within the open-ended questions following the technology vignettes. Specifically, the input provided to the following questions was used: (1) “What initial impressions do you have when thinking about (using an accelerometer/receiving text messages) after a hospital stay?” and (2) “What questions come to mind that you would want answered before agreeing to (wear an accelerometer/answering questions from your care team via text message) after a hospital stay?” Keywords were derived from responses via close examination of responses, whereby recurring patterns and terms arose. Coding was performed to capture core messages provided by survey respondents, allowing the data to be transformed into generalizable, theoretical elements related to the main research questions. Design themes were derived from coded values, whereby design principles were created via the group’s conceptualization of keywords, codes, and themes. The final design principles are shared as the final product to help inform the design of future technological tools to support patients transitioning from hospital to home. This process was conducted independently by 2 individuals trained in research methods with the following characteristics: Analyst A is a first-generation Asian American woman with training in public health science and epidemiology with extensive experience within health care delivery systems, while Analyst B is a White woman with training in human-centered design with experience in designing new products and services to enhance patient experiences with health care. Analyst A’s experience navigating systemic barriers in health care for older family members informed her sensitivity to issues of access and equity, whereas Analyst B’s 20-year experience designing digital solutions shaped her sensitivities to digital barriers. While Analyst A shares lived experience as a caregiver to the study population, she remains mindful of the varying challenges and psychosocial factors that patients of different ages, socioeconomic levels, and cultural backgrounds may face upon hospital discharge, thereby allowing her to approach data with a mindset open to understanding different perspectives. Analyst B, as a trained designer who is practiced in contextual inquiry and eliciting people’s behaviors, goals, and motivations, approached this work with a trained perspective of understanding patient needs through qualitative research methods and analysis. The collaboration was grounded in mutual respect and critical dialogue, allowing us to challenge each other’s assumptions and enrich our interpretations. The team recognized that positionalities are not fixed and committed to ongoing reflexivity throughout the research process.

### Analysis of Quantitative Survey Responses

Quantitative survey response data were used to reinforce the design principles derived via our qualitative approach and were descriptive. Likert-based survey responses are described using frequency (n) and proportion. All data management and analyses were performed using SAS (version 9.2; SAS Institute). Response visualizations were created using R statistical software (version 4.1.2; R Foundation for Statistical Computing) and the ggplot2 package.

### Ethical Considerations

This study was reviewed by the Mayo Clinic Institutional Review Board and deemed exempt from Institutional Review Board approval under 45 CFR 46.104(d), category/subcategory 2(i) (24‐005650; principal investigator LMP). Confidential or identifying information was not collected for study participants, and all data were shared in aggregate form. Remuneration for study participation was not provided. As protected health information was not requested, written consent and Health Insurance Portability and Accountability Act (HIPAA) authorization were not required following 45 CFR 160.103.

## Results

### Overview

We had 116 completed responses to our vignette-based investigation (116/967, 12% response rate; excluding 33 bounced emails; Table 1). Our responding population had a median age of 71 (IQR 61-78) years, was predominantly of White race (112/116, 97%), and reported having chronic obstructive pulmonary disease (103/116, 88.8%), diabetes (87/116, 75.0%), cancer (65/116, 56.0%), osteoarthritis/chronic joint pain (63/116, 54.3%), and predominantly “good” (48/116, 41.4%) or “fair” (29/116, 25.0%) health. Most respondents reported having access to a smartphone (98/116, 84.5%), personal computer/laptop (85/116, 73.3%), and in-home internet (111/116, 95.7%).

**Table 1.** Descriptive characteristics of respondents to vignette-based survey regarding technology to support hospital discharge (N=116).

Characteristic	Overall
Age (years), median (IQR)	71 (61-78)
Race, n (%)	
Black, African, or African American	1 (0.9%)
White	112 (96.6%)
Mixed races	1 (0.9%)
Prefer not to disclose	1 (0.9%)
Missing	1 (0.9%)
Comorbidities, n (%)	
Chronic obstructive pulmonary disease	103 (88.8%)
Diabetes (type 1 or type 2)	87 (75.0%)
Cancer	65 (56.0%)
Osteoarthritis/chronic joint pain	63 (54.3%)
High cholesterol	57 (49.1%)
Hypertension/high blood pressure	42 (36.2%)
Self-rated health, n (%)	
Excellent	3 (2.6%)
Very good	26 (22.4%)
Good	48 (41.4%)
Fair	29 (25.0%)
Poor	10 (8.6%)
Technology access (select all that apply), n (%)	
Smartphone	98 (84.5%)
Personal computer/laptop	85 (73.3%)
Tablet	47 (40.5%)
Smartwatch	14 (12.1%)
None	1 (0.9%)
Are you able to access the internet from your smartphone when you are not at home or logged into an internet/Wi-Fi service?, n (%)	
The patient does not have a smartphone	18 (15.5%)
Yes	88 (89.8%)
No	8 (8.2%)
I do not know	2 (2.0%)
Do you access the internet from your home?, n (%)	
Yes	111 (95.7%)
No	4 (3.4%)
I do not know	1 (0.9%)
Are you satisfied with your ability to access the internet?, n (%)	
Patient does not have in-home internet	5 (4.31)
Satisfied	91 (82.0%)
Neutral	18 (16.2%)
Dissatisfied	2 (1.8%)

### Themes Associated With Technology-Supported Hospital Discharge

Several themes arose during qualitative analysis. The themes are outlined below with representative patient quotes.

#### Connection and Response

Most participants felt positively toward having a continuous connection with Mayo Clinic once discharged and thought they would benefit from sharing their data and receiving recommendations or other support to manage their health. One respondent wrote,

If it helps my providers gain needed information that can improve my overall outcome, I am in favor.

while another stated,

I really like the idea. It would be able to track how a person is generally doing instead of relying 100% on the patient.

#### Technical Ease of Use

Participants felt comfortable with the technology associated with these concepts, although some expressed concerns with management regarding difficulties if the technology proved challenging. One participant stated,

I am afraid I wouldn’t understand how to use the device or if there was a glitch, I wouldn’t be able to figure out how to fix it.

and another less tech-savvy patient mentioned wanting “an alternative way to keep in touch because my techno skills are non-existent.”

#### Solution Usability

Participants had questions or concerns with the wearability, simplicity, or frequency of use/interaction associated with the solution. This was the most popular theme, as participants recognized the impact of daily use. Several participants were wondering how long they would need to wear the device. One asked,

Do I wear it 24 hours a day?

Regarding simplicity, another stated,

I don’t look at my phone much sometimes 1 time a day.

suggesting they may need to change their behavior if more frequent interaction is needed.

#### Human Connection

Participants mentioned a concern that these technologies could take away from the human connection, replacing it with an impersonal solution that might not recognize their specific needs. One participant mentioned,

It’s okay but I feel it is better to talk to someone so I can express my feelings about my recovery.

and another stated,

I would not want these text messages to replace the after-hospital discharge phone call from a hospital staff member.

#### Cost

Participants were concerned about any costs associated with the product and expected not to incur any extra costs. One participant summed up the general feelings of the group by stating,

I would not expect to be charged for the device or access.

#### Privacy and Security

Participants were concerned about data privacy and the security of the information shared. One participant mentioned,

What exactly does it monitor and who has access to the data?

Another stated, “I would want to make sure it is the doctor and not spam,” and still another mentioned, “it seems a bit invasive.”

### Patient-Reported Technology Attribute Importance to Support Hospital Discharge

**Figure 2.**
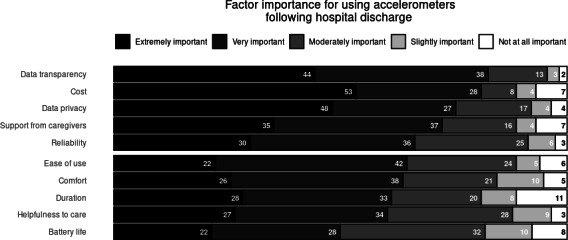
Factor importance for using accelerometers following hospital discharge.

Regarding use of an accelerometer following a hospital stay, respondents reported that “the ability to see my own accelerometer data” (95/116, 81.9% rated as “Very important” or “Extremely Important”) and “how much I would need to pay” (94/116, 81.0% rated as “Very Important” or “Extremely Important”) had the highest cumulative levels of importance impacting willingness to use an accelerometer following a hospital stay. These were followed closely by “reassurance that my data is private between me and my provider team” (87/116, 75% rated as “Very Important” or “Extremely Important”) and “how supportive my caregivers are to my use” (84/116, 72.4% rated as “Very Important” or “Extremely Important”). Respondents noted that “how long I would need to wear an accelerometer” (22/116, 19% rated as “Not Important at all” or “Slightly Important”) and “How long the battery would last” (21/116, 18.1% rated as “Not Important at all” or “Slightly Important”) were cumulative of least importance regarding willingness to use the device (Figure 2).

**Figure 3.**
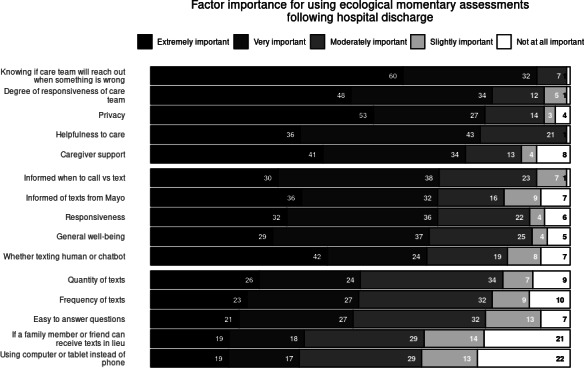
Factor importance for using ecological momentary assessments following hospital discharge.

When asked about factor importance in respondents’ willingness to receive ecological momentary assessments following a hospital stay, the factors with the highest cumulative ranked importance were: “Whether a member of the care team would reach out if something was wrong” (107/116, 92.2% rated as “Very Important” or “Extremely important”), “How responsive the care team is to my answers” (95/116, 81.9% rated as “Very Important” or “Extremely important”), and “How helpful I feel the questions will be to my care” (92/116, 79.3% rated as “Very Important” or “Extremely important”; Figure 3). Respondents indicated “Whether I could have a family member or friend receive the messages instead of me” (24/116, 20.7% rated as “Not at all Important”) and “Whether I can use my computer or tablet to answer instead of a cell phone” (25/116, 21.6% rated as “Not at all Important”) had most responses indicating this factor was of no importance at all.

### Design Principles for Technology-Supported Hospital Discharge

**Figure 4.**
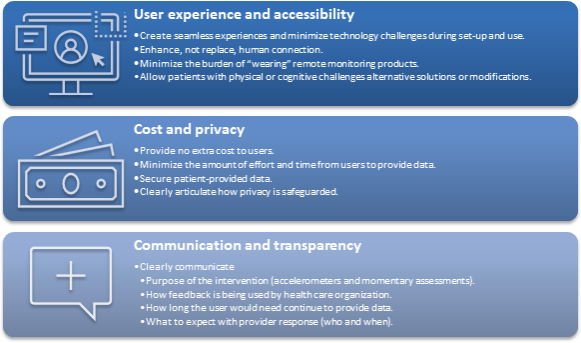
Design principles for technology to support patients during hospital discharge.

Using the themes as guidance, 3 design principles were derived to highlight how concepts should perform (Figure 4). The first design principle describes user experience and accessibility by patients and includes constructs such as a seamless experience for end users (patients) during initial engagement with and use of the technology, how technology support should enhance the connection patients feel to care teams and not replace the human connection, how technology needs to minimize the amount of effort needed by patients to enable the end goal of supportive healing at home following hospital discharge, and how modifications are needed to support patients with physical or cognitive limitations, such as patients reliant on wheelchairs or caregivers for cognitive support.

The second design principle derived focuses on consideration of cost to the patient when deploying new technology-based support for patients transitioning from hospital to home care. Cost is broadly defined as financial, including copayments and out-of-pocket costs, in addition to time and effort needed to participate in postdischarge, technology-based care models. Patients also identify the need for data security to ensure their information is private between the patient and the care team, and clear communication on how data privacy will be assured.

The third design principle emphasizes the need for communication about the purpose of the intervention and how the technology will be part of their care experience and used by the care team. Expectations regarding the duration of expected engagement with the technology were also important to patients, as was a clear understanding of which member of the care team would be interacting with the technology, and what the anticipated response content and timeline may be.

## Discussion

### Principal Findings

In this study, we derived 3 categories of design principles to aid in the design, development, and deployment of technology to support patients as they transition from hospital to home. We observed that patients value technology fitting seamlessly into their care experience, extending but not replacing human connection. Within our study, nearly every respondent indicated high importance that care teams be responsive to information received via technology, particularly if something seems to be going wrong. Patients were sensitive to additional costs that could be incurred based on use of these new technologies and placed high importance on data privacy and security in their willingness to engage with technology to support hospital discharge. Communication and transparency of the “why, what, who, and when” is important when engaging with technology to support transitions from hospital care to home. While the technical aspects of using technology are a consideration for patients, battery life, duration of use, and ability to use alternative mechanisms such as tablets or desktop computers for participation were of lower importance in patients’ willingness to engage.

### Comparison With Previous Work

Several studies have demonstrated a high degree of acceptance of digital technology to support hospital-to-home transition [7,9,27,28]. Studies deploying SMS-based text messaging had high patient satisfaction rates, and patients rated programs high on convenience and responsiveness during hospital-to-home transitions [7,27]. In addition, patients have reported appreciation for the flexibility enabled via automation and the asynchronous nature of technology solutions compared with traditional in-person care models [29]. Within our design exercise, we observed that patients value human connection supplemented by, but not replaced by, technology, building upon these aspects reported in the literature. Technology supporting human connection has been demonstrated in the hosting of virtual birthdays, funerals, and graduations, enabling shared human experiences across physical distances, which is particularly helpful for individuals experiencing physical disabilities, frailty, or limited resources to support travel. In health care, technology has enabled human connection to mental health support that is accessible and practical, particularly for certain populations, such as adolescents [30] and those residing in areas with decreased availability of support services [31]. The multimodal delivery of mental health support, including video- and telephone-enabled care, mobile apps, and therapeutic gaming, supports the ability to foster human connection via technology [32].

Research has shown that bidirectional communication is considered more valuable than automated reminders by patients [9,19]. Both within our study and as reported elsewhere [33], patients indicate the desire to know that the interactions they are having with a device are being monitored or overseen by a human being, with the ability to escalate needs should a patient’s condition warrant increased levels of care [34]. This reassurance has been reported to increase patient and caregiver confidence in the transition from institutionalized care to home care [34]. Use of technology to support hospital-to-home transitions is a mechanism to mitigate costs experienced by health care systems and payers alike via improved patient outcomes and decreased use [7,35]. However, the technology needed to support transitions from hospital to home may consist of personal computers, laptops, or tablets for inputting information, and in-home monitoring supplies such as glucose or blood pressure monitoring. Payment models to support these costs vary greatly, from coverage by payers to hospital systems to patients and families themselves. In addition to being cognizant of the costs associated with these new technologies, health care institutions need to address patient privacy and communicate those privacy considerations to patients upon consent for participation. Devices and platforms must be chosen that follow federal requirements (ie, HIPAA compliance within the United States), which require end-to-end data encryption, access controls, and audit trails. In addition, health care systems must require role-based access controls to ensure that only pertinent members of the care team can access patient information. All of these requirements must be supported by robust informed consent processes that ensure that patients understand how their data will be collected, used, and shared, and how patients and caregivers can protect themselves via secured network access, locking of personal devices, and avoidance of phishing.

The ability to incorporate supportive technology into transitions from hospital-based care to home as part of the care team, and not a separate entity, was emphasized as a need by our study participants. Technologies such as SMS-based text messaging, accelerometers, and other tools could be used to facilitate seamless transitions between our traditional care settings of hospital-based care, postacute care, and home with return to primary care. Patients with complex medical needs can experience frequent care transitions between our traditional care settings. However, care must be taken to ensure that safe and effective care is being maintained, particularly among those in a medically vulnerable state, such as following hospital discharge. Accuracy of supportive technology must be ensured so that care teams responding to in-home monitoring have reliable information, and devices must be usable by patients and caregivers. One opportunity is the movement toward “Bring Your Own Device” to help ensure usability; however, variability exists in the accuracy of direct-to-consumer monitoring devices, such as for blood pressure [36]. Health care systems may opt to designate a set of acceptable monitoring devices, allowing patients to use the device they choose within safety parameters outlined by health care providers. Grounding new tools in user experience and accessibility, cost and privacy, and communication and transparency can help clinical teams and developers improve the incorporation of tools into routine clinical care, thereby advancing seamless transitions during an anxiety-filled and uncertain time for patients.

### Strengths and Limitations

Our study had limitations. Although our sample would be considered large for a qualitative assessment (n=116), the overall response rate was low (12%). We believe that lack of incentivization for study participation, use of patient-provided email as opposed to use of the clinic-provided patient portal, and survey availability in a single language (English) may have contributed to our low overall response rate. In addition, our response population was predominantly older adults (median age 71 years, IQR 61-78) and White (112/116, 97%) respondents from a single academic medical center, which may limit our ability to generalize to other populations. The older age of our participants may have influenced the digital literacy and access of our population, as research shows that older adults are more likely to experience decreased access to digital tools and services [37], and decreased digital literacy serves as an independent stressor to the well-being of older adults [38]. In addition, individuals of racial and ethnic minority groups are less likely to feel trust in the health care system and may be less willing to provide surveillance data directly back to their health care provider. Our response population was generally favorable about the use of movement tracking and text-based assessments following hospital discharge, but these results may not be consistent with other populations. Our investigation focused on 2 technologies (ecological momentary assessments via text message and accelerometers), which may not encompass the nuances of new tools that could be created to support all care transitions. One of the tools explored (accelerometers) is considered an emerging technology, which may not be validated for use in clinical settings. Our study had several strengths. First, this is one of the few investigations published to incorporate design thinking and UCD practices to help inform the design, development, and implementation of technology to support patients transitioning from hospital to home, focusing on the practical implications of new tools or services. Second, we used a vignette-based, mixed methods inquiry to derive open-ended feedback from a large qualitative sample of individuals who recently experienced hospital-to-home discharge. We used qualitative methodology to derive design constructs that can be used in multiple design settings and validate findings with quantitative assessment derived through the face validation exercise of patient interviews.

### Future Directions

As health care systems increasingly rely on digital technologies to support transitions from hospital to home, future research must prioritize equity, inclusivity, and adaptability, building on and testing the design principles presented here. One critical area for exploration is how these design principles perform across diverse cultural, racial, and demographic groups, given the limited diversity in our present sample. In addition, opportunities exist to evaluate emerging technologies—such as artificial intelligence–driven care coordination platforms, wearable sensors, and voice-assisted devices—for their usability, accessibility, and clinical impact. These innovations hold promise for improving postdischarge outcomes, but their success depends on aligning with patients’ real-world needs, preferences, and capabilities. Finally, interdisciplinary collaboration among clinicians, social scientists, caregivers, and patients themselves will be essential to design and implement user-centered, culturally responsive digital solutions. By addressing these gaps, future work can help ensure that digital health technologies truly enhance recovery and reduce disparities as patients transition from hospital to home.

### Conclusion

Our study identified key design principles for developing technology to support patients transitioning from hospital to home, emphasizing the importance of seamless integration into care, minimizing patient and caregiver burden, and ensuring data privacy and security. Patients value human connection supplemented by technology and prefer transparent communication about the use and purpose of these tools. Future efforts should focus on incorporating these principles to enhance patient experience and outcomes during postacute care transitions.

## Supplementary material

10.2196/72913 Multimedia Appendix 1 Semistructured interview guide.

10.2196/72913 Multimedia Appendix 2 Deployed survey instrument.

10.2196/72913 Checklist 1 Checklist for Reporting Results of Internet E-Surveys (CHERRIES) checklist.

## Abbreviations:

CHERRIES: Checklist for Reporting Results of Internet E-Surveys
EQ-5D: EuroQol 5-Dimension Scale
HIPAA: Health Insurance Portability and Accountability Act
TAM: technology acceptance model
UCD: user-centered design
