# Supplementary material for: Patient-Derived Design Principles for Technology-Enabled Healing at Home Following Hospital Discharge: Mixed Methods Study
Source: JMIR Hum Factors. 2025 Aug 20;12:e72913. doi: 10.2196/72913 (PMC12367353; doi:10.2196/72913)
Supplement: Multimedia Appendix 1 [file humanfactors-v12-e72913-s001.docx]

**Interview Guide SGP:**

***“Thank you for taking the time to share today. My name is [name], and I will be conducting your interview today. Today’s interview will last about 30 minutes.”***

***“Today we are going to be talking about technologies that Mayo Clinic is thinking about using to learn how our patients are recovering after a stay in a Mayo Clinic hospital. We will talk about two different types of technology that may be used to learn how patients are doing after they leave the hospital. I will explain the two technologies and then ask you a few questions to get your thoughts on both. Our main goal today is to hear about any questions you have with the technology, how we explain the technology, or the questions we ask about the technology. We will use your feedback to edit our text before sending out to a larger group of patients for feedback.”***

***“Before we begin, I want to go over a few things. We are recording the session because we don’t want to miss any of your comments. Please speak up so we can capture what you are saying. We’ll be on a first name basis, and in our later reports there will not be any names attached to comments. You may be assured of confidentiality. What is said in the room stays in the room.”***

***“My role here is to ask questions and listen. I won’t be participating in the conversation. I will explain technology and then we will go over a few questions. I’ll be moving the discussion from one question to the next. There are no right or wrong answers, all ideas, experiences and opinions are valuable. You can skip any questions or comments that you would not like to answer."***

***“Are you interested in continuing with our conversation today?”***

***“Do you consent to me recording our discussion?”***

**VIGNETTE 1:**

**The first technology is an Accelerometer. An accelerometer can detect your physical activity and movement as you go about your day without any action from you. Accelerometers can be found in smartphones, smartwatches (Apple Watch, Galaxy, Garmin), and other small devices that you would have on your body. We want to learn how you’re moving and how much you are moving to help your care team make sure you are getting better after being in the hospital.**

**An accelerometer would be worn all the time for up to one month after you leave the hospital. You are able to wear the device while you are washing your hands, showering, bathing, etc. The only time you would need to take it off is if you were in a deep pool or lake as the device cannot be submerged in water.**

1. **Did the description of the accelerometer I provided make sense to you?**
2. **Do you have any questions about what an accelerometer is??**
   1. **If yes, answer questions**
   2. **If no, proceed to question 2**
3. **I mentioned that an accelerometer is a device found in smartphones, smartwatches, or other small devices that you would have on your body. Does that description make sense to you? Are there other details that we should provide to people to help them understand what this technology could be?**
4. **I mentioned that our care teams at Mayo Clinic would be using the accelerometer that you wear to see how well you are moving after leaving the hospital. This would help our teams to know that you are healing well. Does that rationale make sense to you, or what questions might you have about how we would use this information?**
5. **Please share any thoughts or concerns you would like to share with the team about your feelings on wearing the accelerometer after you leave the hospital.**

**“Any final comments regarding this first scenario you would like to share before moving on to our second scenario?”**

**VIGNETTE 2:**

**“Ecological Momentary Assessments” are questions that your care team sends to you in a text message to your cell phone. These messages could ask about things like being able to schedule an appointment with your provider, if you were able to get your prescriptions filled or to see how you are feeling after leaving the hospital. Your answers to these questions can help your care team know if you need help with tasks your provider wants you to do after your appointment, or to let your care team know that you are doing OK.**

1. **Overall, did the description I just provided to you make sense? What questions might you have about ecological momentary assessments?**
2. **I mentioned that your care team may send you messages about topics like your ability to schedule an appointment with your doctor, or whether you were able to get your prescriptions filled after you left the hospital. Do those examples make sense to you?**
3. **I mentioned that your care team would use your answers to these messages to makes sure you are getting what you need after your hospital stay. Did that come across to you?**
4. **Please share any thoughts or concerns you would like to share with the team about your feelings on getting text-based questions to your cell phone after you leave the hospital.**

**“Any final comments you would like to share regarding ecological momentary assessments to answer questions following a stay?”**

**“Thank you for your time today in answering these questions. Is there anything else you would like to share with our study team before we complete our time today?”**

**“Again, thank you for your time.”**
