# Supplementary material for: Patient-Derived Design Principles for Technology-Enabled Healing at Home Following Hospital Discharge: Mixed Methods Study
Source: JMIR Hum Factors. 2025 Aug 20;12:e72913. doi: 10.2196/72913 (PMC12367353; doi:10.2196/72913)
Supplement: Multimedia Appendix 2 [file humanfactors-v12-e72913-s002.docx]

10730 PHILPOT LM (Mayo Clinic)

Start of Block: SCREENING QUESTION

| 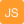 |
| --- |

Q1 **Our records show that you had a recent stay at a Mayo Clinic hospital. Is that correct?**

- Yes (1)
- No (2)
- I don't know (3)

End of Block: SCREENING QUESTION

Start of Block: GENERAL USE OF TECHNOLOGY

Display This Question:

If Q1 = 1

Q2 Today we would like to ask you about technologies we are considering using to help understand how our patients are recovering following a stay in a Mayo Clinic hospital. First, we will ask you a few questions about how you use technology today. Technology could include laptop computers, desktop computers, smartphones, smartwatches, or other technologies. It's OK if you don't use any of these technologies.


 **Do you currently have access to any of the following?**  (Select all that apply.)

- A personal computer or laptop (1)
- A tablet like an iPad, Samsung Galaxy, Motorola Xoom, Kindle Fire, or other (2)
- A smartphone, such as an iPhone, Android, Blackberry, or Windows phone (3)
- A smartwatch, such as Apple Watch, Galaxy, or Garmin (4)
- ⊗None of the above (5)

Display This Question:

If Q2 = 3

| 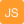 |
| --- |

Q3 **Are you able to access the internet from your smartphone when you are not at home or logged into an internet/wifi service?**

- Yes (1)
- No (2)
- I don't know (3)

Display This Question:

If Q1 = 1

| 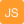 |
| --- |

Q4 **Do you access the internet from your home?**

- Yes (1)
- No (2)
- I don't know (3)

Display This Question:

If Q4 = 1

| 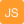 |
| --- |

Q5 **Are you satisfied with your ability to access the internet?**

- Satisfied (1)
- Neutral (2)
- Dissatisfied (3)

| Page Break |  |
| --- | --- |

Display This Question:

If Q1 = 1

Q6 **Do you ever seek internet or computer access at any of the following venues?**  (Select all that apply.)

- School or place of education (1)
- Work or workplace (2)
- Public library (3)
- Church, religious center, or place of worship (4)
- Café or coffee shop (5)
- Community center, youth center, center for senior adults (6)
- Other, please specify: (7) __________________________________________________
- ⊗I do not seek internet or computer access at any venue (8)

Display This Question:

If Q1 = 1

| 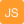 |
| --- |

Q7 **Have you ever taken a class to learn about how to use computers, smartphones, tablets, or other devices?**

- Yes (1)
- No (2)
- I don't know (3)

| Page Break |  |
| --- | --- |

Display This Question:

If Q1 = 1

Q8 **Please select your level of agreement with the following statements.**

|  | Strongly disagree (1) | Disagree (2) | Neutral (3) | Agree (4) | Strongly agree (5) |
| --- | --- | --- | --- | --- | --- |
| When I get a new electronic device, I usually need someone else to set it up or show me how to use it. (1) |  |  |  |  |  |
| Among my peers, I am usually the first to try out new information technologies. (2) |  |  |  |  |  |
| If I needed to send a message to my healthcare team today, I would easily be able to do so. (3) |  |  |  |  |  |
| If I needed to see my doctor using a camera or video today, I would easily be able to do so. (4) |  |  |  |  |  |
| I have used a tablet or smartphone to help me achieve a health-related goal such as monitoring my health conditions, quitting smoking, losing weight, or increasing physical activity. (5) |  |  |  |  |  |
| I would be interested in learning about alternative/new ways to talk with my care team. (6) |  |  |  |  |  |

End of Block: GENERAL USE OF TECHNOLOGY

Start of Block: TECHNOLOGY VIGNETTES

Display This Question:

If Q1 = 1

| 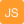 |
| --- |

Q9 **Mayo Clinic is considering whether and how different types of technology could be used to understand how patients are doing when they leave the hospital. We would like to ask your opinions on two different types of technologies.**

 Technology #1: Movement Accelerometers

“Accelerometers” are technologies that can detect your physical activity and movement without requiring any action from you as you go about your day. These non-invasive technologies can be placed within smartphones, smartwatches, and other small devices that you would have on your body. The goal is to understand how you're moving and how much you're moving to help your care team ensure that you are getting better after your hospital stay.


 **Given this information, would you be willing to wear an accelerometer in the form of a traditional wrist-watch after a hospital stay?**

- Yes (1)
- No (2)
- I don't know (3)

Display This Question:

If Q1 = 1

Q10 **What initial impressions do you have when thinking about using an accelerometer after a hospital stay?**

________________________________________________________________

________________________________________________________________

________________________________________________________________

________________________________________________________________

________________________________________________________________

Display This Question:

If Q1 = 1

Q11 **What questions come to mind that you would want answered before agreeing to wear an accelerometer after a hospital stay?**

________________________________________________________________

________________________________________________________________

________________________________________________________________

________________________________________________________________

________________________________________________________________

| Page Break |  |
| --- | --- |

Display This Question:

If Q1 = 1

Q12 **Rate the importance of the following aspects related to your willingness to use an accelerometer following your hospital stay.**

|  | Not at all important (1) | Slightly important (2) | Moderately important (3) | Very important (4) | Extremely important (5) |
| --- | --- | --- | --- | --- | --- |
| How easy the accelerometer would be to use (1) |  |  |  |  |  |
| How comfortable an accelerometer would be to wear (2) |  |  |  |  |  |
| How much I would need to pay for an accelerometer (3) |  |  |  |  |  |
| How helpful I feel an accelerometer would be to my care (4) |  |  |  |  |  |
| How reliable I think an accelerometer would be (5) |  |  |  |  |  |

| Page Break |  |
| --- | --- |

Display This Question:

If Q1 = 1

Q13 **Rate the importance of the following aspects related to your willingness to use an accelerometer following your hospital stay.**

|  | Not at all important (1) | Slightly important (2) | Moderately important (3) | Very important (4) | Extremely important (5) |
| --- | --- | --- | --- | --- | --- |
| Reassurance that my accelerometer data is private between me and my provider team (1) |  |  |  |  |  |
| How supportive my caregivers are to my use of an accelerometer (2) |  |  |  |  |  |
| How long will I need to wear an accelerometer for (3) |  |  |  |  |  |
| The ability to see my own accelerometer data (4) |  |  |  |  |  |
| How long the accelerometer battery would last before I need to recharge it (5) |  |  |  |  |  |

| Page Break |  |
| --- | --- |

Display This Question:

If Q1 = 1

| 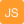 |
| --- |

Q14 **Do you feel that having a device such as an accelerometer might change your motivation to be more active following hospital discharge?**

- Yes (1)
- No (2)
- I don't know (3)

Display This Question:

If Q1 = 1

Q15 **Please provide any other thoughts you’d like to share with the team regarding your willingness to use an accelerometer following a hospital stay.**

________________________________________________________________

________________________________________________________________

________________________________________________________________

________________________________________________________________

________________________________________________________________

| Page Break |  |
| --- | --- |

Display This Question:

If Q1 = 1

| 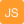 |
| --- |

Q16 Technology #2: Ecological Momentary Assessments and Status Check-Ins

 “Ecological Momentary Assessments,” are questions sent to you as a text message to your cell phone as you go about your typical daily activities. These text messages could ask whether you were able to schedule an appointment with your doctor, whether you were able to fill the medications you were prescribed, or ask about how you are feeling after a hospital stay, as examples. Answers to these questions could help us to know whether you need help with the tasks the doctor asked you to do after your appointment, or to let our doctors know that you are doing ok.


 **Given this information, would you be willing to answer questions via text message sent to your cellular phone following a stay in the hospital?**

- Yes (1)
- No (2)
- I don't know (3)

Display This Question:

If Q1 = 1

Q17 **What are your thoughts on receiving text messages from your care team?**

________________________________________________________________

________________________________________________________________

________________________________________________________________

________________________________________________________________

________________________________________________________________

Display This Question:

If Q1 = 1

Q18 **What would you want to know before answering questions from your care team via text message?**

________________________________________________________________

________________________________________________________________

________________________________________________________________

________________________________________________________________

________________________________________________________________

| Page Break |  |
| --- | --- |

Display This Question:

If Q1 = 1

Q19 **Rate the importance of the following aspects related to your willingness to receive questions via text message following a hospital stay.**

|  | Not at all important (1) | Slightly important (2) | Moderately important (3) | Very important (4) | Extremely important (5) |
| --- | --- | --- | --- | --- | --- |
| How easy the questions are to answer (1) |  |  |  |  |  |
| Whether I am texting with a human or a machine/chatbot (2) |  |  |  |  |  |
| Whether I am told that I will be receiving text messages from Mayo (3) |  |  |  |  |  |
| Whether I can use my computer or tablet to answer instead of a cell phone (4) |  |  |  |  |  |
| Whether I would receive a response to my text messages (5) |  |  |  |  |  |

| Page Break |  |
| --- | --- |

Display This Question:

If Q1 = 1

Q20 **Rate the importance of the following aspects related to your willingness to receive questions via text message following a hospital stay.**

|  | Not at all important (1) | Slightly important (2) | Moderately important (3) | Very important (4) | Extremely important (5) |
| --- | --- | --- | --- | --- | --- |
| How sick I am feeling (1) |  |  |  |  |  |
| Whether I could have a family member or friend receive the messages instead of me (2) |  |  |  |  |  |
| How helpful I feel the questions would be to my care (3) |  |  |  |  |  |
| How many text messages I would receive (4) |  |  |  |  |  |
| Whether I know when to text message my care team versus call them (5) |  |  |  |  |  |

| Page Break |  |
| --- | --- |

Display This Question:

If Q1 = 1

Q21 **Rate the importance of the following aspects related to your willingness to receive questions via text message following a hospital stay.**

|  | Not at all important (1) | Slightly important (2) | Moderately important (3) | Very important (4) | Extremely important (5) |
| --- | --- | --- | --- | --- | --- |
| How often I would receive text messages (1) |  |  |  |  |  |
| How responsive the care team is to my answers (2) |  |  |  |  |  |
| Whether a member of the care team would reach out if something was wrong (3) |  |  |  |  |  |
| Reassurance that the text messages are private between me and my care team (4) |  |  |  |  |  |
| How supportive my caregivers are to my use of text messages to my care team (5) |  |  |  |  |  |

| Page Break |  |
| --- | --- |

Display This Question:

If Q1 = 1

Q22 **Please provide any other thoughts you’d like to share with the team regrading your willingness to receive text-based questions to your cellular phone following a hospital stay.**

________________________________________________________________

________________________________________________________________

________________________________________________________________

________________________________________________________________

________________________________________________________________

End of Block: TECHNOLOGY VIGNETTES

Start of Block: HEALTH STATUS AND DEMOGRAPHICS (EQ-5D)

Display This Question:

If Q1 = 1

| 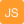 |
| --- |

Q23 Finally, we hope to ask a few final questions about you and your heath.

 The following questions ask you about your overall health and abilities.

 Under each group, please select the ONE response that best describes your own state of health TODAY.     **MOBILITY**1

- I have no problems in walking about (1)
- I have some problems in walking about (2)
- I am confined to bed (3)

Display This Question:

If Q1 = 1

| 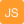 |
| --- |

Q24 **SELF-CARE**1

- I have no problems with self-care (1)
- I have some problems washing or dressing myself (2)
- I am unable to wash or dress myself (3)

| Page Break |  |
| --- | --- |

Display This Question:

If Q1 = 1

| 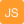 |
| --- |

Q25 **USUAL ACTIVITIES** *(e.g., work, study, housework, family, or leisure activities)*1

- I have no problems with performing my usual activities (1)
- I have some problems with performing my usual activities (2)
- I am unable to do my usual activities (3)

Display This Question:

If Q1 = 1

| 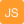 |
| --- |

Q26 **PAIN / DISCOMFORT**1

- I have no pain or discomfort (1)
- I have moderate pain or discomfort (2)
- I have extreme pain or discomfort (3)

Display This Question:

If Q1 = 1

| 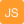 |
| --- |

Q27 **ANXIETY / DEPRESSION**1

- I am not anxious or depressed (1)
- I am moderately anxious or depressed (2)
- I am extremely anxious or depressed (3)

Display This Question:

If Q1 = 1

| 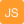 |
| --- |

Q28 **In general, would you say your health is:**

- Excellent (1)
- Very good (2)
- Good (3)
- Fair (4)
- Poor (5)

| Page Break |  |
| --- | --- |

Display This Question:

If Q1 = 1

Q29 **Has a doctor or other healthcare professional ever told you that you have any of the following medical conditions?**

|  | Yes (1) | No (2) | I don't know (3) |
| --- | --- | --- | --- |
| Cancer (1) |  |  |  |
| High Cholesterol (2) |  |  |  |
| Hypertension or High Blood Pressure (3) |  |  |  |
| Diabetes (Type 1 or Type 2) (4) |  |  |  |
| Chronic Obstructive Pulmonary Disease (5) |  |  |  |
| Osteoarthritis or Chronic Joint Pain (6) |  |  |  |

| Page Break |  |
| --- | --- |

Display This Question:

If Q1 = 1

Q30 **Which race(s) do you most closely identify?**

- Asian, South Asian, or Asian Pacific (1)
- Black, African, or African American (2)
- Central or South American (3)
- Mexican or Mexican American (4)
- Middle Eastern (5)
- Native American (6)
- Native Hawaiian or Pacific Islander (7)
- White American, Caucasian, or White European (8)
- Mixed race(s) (9)
- ⊗None of these (10)
- ⊗Prefer not to answer (11)

Display This Question:

If Q1 = 1

| 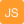 |
| --- |

Q31 **How comfortable would you say you are reading in English?**

- Uncomfortable (1)
- Neutral (2)
- Comfortable (3)

Display This Question:

If Q1 = 1

| 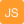 |
| --- |

Q32 **How comfortable would you say you are speaking in English?**

- Uncomfortable (1)
- Neutral (2)
- Comfortable (3)

| Page Break |  |
| --- | --- |

Display This Question:

If Q1 = 1

Q33 **Please let the research team know of any other thoughts or comments you have related to this study:**

________________________________________________________________

________________________________________________________________

________________________________________________________________

________________________________________________________________

________________________________________________________________

End of Block: HEALTH STATUS AND DEMOGRAPHICS (EQ-5D)

Start of Block: Submit

Display This Question:

If Q1 = 1

Q34 Reference
 1Rabin, R., & de Charro, F (2001). EQ-5D: a measure of health status from the EuroQol Group. Annals of Medicine 33(5):337-343.

     THANK YOU FOR COMPLETING THE SURVEY!

 **Please click SUBMIT to record your answers.**

Display This Question:

If Q1 = 2

Or Q1 = 3

Q35   Thank you for your interest in this study.
**Please click SUBMIT to end the survey.**

End of Block: Submit
